# Development and validation of a nomogram for postoperative sleep disturbance in adults: a prospective survey of 640 patients undergoing spinal surgery

**DOI:** 10.1186/s12871-023-02097-x

**Published:** 2023-05-04

**Authors:** Jin Du, Honggang Zhang, Zhe Ding, Xiaobin Wu, Hua Chen, Weibin Ma, Canjin Qiu, Shengmei Zhu, Xianhui Kang

**Affiliations:** 1 Department of Anesthesiology, the First Affiliated Hospital, Zhejiang University School of Medicine, Hangzhou, China; 2 Department of Anesthesiology, China Coast Guard Hospital of the People ’ s Armed Police Force, Jiaxing, China

**Keywords:** Nomogram, Regression analysis, Risk factor, Sleep disturbance, Spinal surgery, Survey

## Abstract

**Background:**

Postoperative sleep disturbance (PSD) is a prevalent clinical complication that may arise due to various factors. The purpose of this investigation is to identify the risk factors for PSD in spinal surgery and establish a risk prediction nomogram.

**Methods:**

The clinical records of individuals who underwent spinal surgery from January 2020 to January 2021 were gathered prospectively. The least absolute shrinkage and selection operator (LASSO) regression, along with multivariate logistic regression analysis, was employed to establish independent risk factors. A nomogram prediction model was devised based on these factors. The nomogram’s effectiveness was evaluated and verified via the receiver operating characteristic (ROC) curve, calibration plot, and decision curve analysis (DCA).

**Results:**

A total of 640 patients who underwent spinal surgery were analyzed in this investigation, among which 393 patients experienced PSD with an incidence rate of 61.4%. After conducting LASSO regression and logistic regression analyses using R software on the variables in training set, 8 independent risk factors associated to PSD were identified, including female, preoperative sleep disorder, high preoperative anxiety score, high intraoperative bleeding volume, high postoperative pain score, dissatisfaction with ward sleep environment, non-use of dexmedetomidine and non-use of erector spinae plane block (ESPB). The nomogram and online dynamic nomogram were constructed after incorporating these variables. In the training and validation sets, the area under the curve (AUC) in the receiver operating characteristic (ROC) curves were 0.806 (0.768–0.844) and 0.755 (0.667–0.844), respectively. The calibration plots indicated that the mean absolute error (MAE) values in both sets were respectively 1.2% and 1.7%. The decision curve analysis demonstrated the model had a substantial net benefit within the range of threshold probabilities between 20% and 90%.

**Conclusions:**

The nomogram model proposed in this study included eight frequently observed clinical factors and exhibited favorable accuracy and calibration.

**Trial registration:**

The study was retrospectively registered with the Chinese Clinical Trial Registry (ChiCTR2200061257, 18/06/2022).

## Background

Postoperative sleep disturbance (PSD) is a prevalent yet often overlooked perioperative complication that has a negative impact on patient sleep quality and recovery. Several previous investigations have reported the incidence of PSD ranging from 30 to 80% [1–3]. Even after six months of serious illness, the incidence rate may still range from 10 to 61% [4]. PSD is generally characterized by sleep fragmentation, deprivation, and a reduction in slow-wave sleep (SWS) and rapid eye movement (REM) sleep, which are quantified by polysomnography. Patients often experience a decline in both the quantity and quality of sleep, an increased tendency to be aroused, and nightmares. Continued sleep deprivation can cause hyperalgesia, heightened stress levels, and negative emotions, all of which can impair patient rehabilitation and prolong hospitalization periods [5–8]. According to a meta-analysis, sleep disturbances were found to have a significant correlation with chronic postsurgical pain [9]. Another investigation revealed that sleep disorders might enhance the likelihood of falls and reduce bone density [10]. Numerous other studies have demonstrated that perioperative sleep disturbances have a detrimental impact on both short-term and long-term patient outcomes [11–13].

During typical spinal procedures like spinal canal decompression, internal fixation of spinal fractures, and scoliosis correction, the likelihood of PSD may increase due to the impact of significant risk factors such as trauma, postoperative pain, perioperative anxiety, advanced age, and so on. [14–17]. Currently, there is no straightforward and precise tool available to forecast the probability of PSD in patients undergoing spinal surgery. Consequently, our aim was to investigate the risk factors for PSD in spinal surgery and establish a dependable risk prediction nomogram. By doing so, we could have a practical and efficient approach to assess the exact likelihood of PSD. This would enable us to identify patients who are at high risk of developing PSD and create a treatment plan in advance. Timely intervention and effective treatment may potentially enhance the postoperative prognosis of sleep quality for such patients.

## Methods

### Study design

As a prospective observational cohort study, no interventions were performed during surgery or general anesthesia. The Research Ethics Committee of China Coast Guard Hospital of People’s Armed Police Force approved this study on November 19, 2019 (Hailun, 2,019,097). All patients willingly participated in the study and provided informed consent before undergoing surgery. The study adhered to the principles of the Declaration of Helsinki and was registered with the Chinese Clinical Trial Registry (ChiCTR2200061257, 18/06/2022).

### Settings and samples

Patients who underwent spinal surgery at our hospital from January 2020 to January 2021 were recruited for this study. The inclusion criteria were as follows: (1) patients undergoing internal fixation, decompression, or orthopedic surgery of the thoracic, lumbar, or cervical spine; (2) patients aged 18 years or older; and (3) patients with American Society of Anesthesiologists (ASA) physical health status grade I-III. The exclusion criteria were: (1) patients who had recently taken sedatives, antidepressants, or anti-anxiety medications in the last week; (2) patients with severe perioperative complications who could not participate in the study; (3) patients admitted to the intensive care unit (ICU) postoperatively; and (4) patients who were unable to cooperate with the investigations due to impaired consciousness or mental disorders.

The sample size was calculated based on prior research and pre-experiments, with an expected positive rate of approximately 65% and 10 ~ 13 factors entering the regression analysis. Taking into account a sample loss rate of 10% ~ 20%, the required sample size for this study was calculated to be no less than 13 × 10 ÷ 0.35 × (1 + 0.2) ≈ 446 cases.

### Outcome variable

The Pittsburgh Sleep Quality Index (PSQI) score was utilized to assess whether the patients had PSD. This questionnaire is a validated and user-friendly tool for evaluating the sleep quality of patients. It consists of 18 scoring items in seven domains, including sleep latency, sleep duration, habitual sleep efficiency, subjective sleep quality, use of sleeping medication, sleep disturbances, and daytime dysfunction. The total score ranges from 0 to 21, with higher scores indicating more severe sleep disorders. In this study, the PSQI questionnaire was administered 1 week postoperatively, and patients with a score above 7 were deemed to have PSD. The outcome variable was evaluated and recorded by designated researchers who were not involved in collecting any other explanatory variables to reduce researcher bias.

### Explanatory variables

Potential predictive factors were selected based on previous literature reviews, clinical experience, and pre-experiments. These included baseline data, medical history, laboratory findings, intraoperative treatment, and questionnaire survey results, among others. Ultimately, 11 continuous variables and 19 dichotomous variables were chosen as explanatory variables, as indicated in Table 1.

**Table 1:** Participant characteristics of the training set and the validation set

Variables	Training set (n = 512)	Validation set (n = 128)	P-value
PSD			
No	198 (38.7%)	49 (38.3%)	0.935
Yes	314 (61.3%)	79 (61.7%)	
Age	62.35 ± 7.72	63.14 ± 6.77	0.286
BMI	23.69 ± 3.26	23.62 ± 3.23	0.851
Operation time	88.63 ± 22.62	88.55 ± 21.62	0.972
Bleeding volume	400(200)	400(150)	0.456^*^
Rehydration volume	1300(400)	1300(500)	0.288^*^
Midazolam	3(1)	3(1)	0.631^*^
Sufentanil	45(10)	40(10)	0.401^*^
PACU residence time	50(20)	50(10)	0.403^*^
CRP	15(7)	15.5(7)	0.956^*^
VAS score	3(2)	3(2)	0.899^*^
SAS score	47.72 ± 10.54	48.17 ± 10.92	0.669
Gender			
Male	283 (55.3%)	69 (53.9%)	0.781
Female	229 (44.7%)	59 (46.1%)	
ASA grade			
<III	457 (89.3%)	118 (92.2%)	0.326
≥III	55 (10.7%)	10 (7.8%)	
Hypertension			
No	292 (57%)	75 (58.6%)	0.749
Yes	220 (43%)	53 (41.4%)	
Diabetes mellitus			
No	397 (77.5%)	104 (81.2%)	0.362
Yes	115 (22.5%)	24 (18.8%)	
Coronary heart disease			
No	481 (93.9%)	123 (96.1%)	0.345
Yes	31 (6.1%)	5 (3.9%)	
Cerebral infarction			
No	486 (94.9%)	121 (94.5%)	0.858
Yes	26 (5.1%)	7 (5.5%)	
OSAS			
No	487 (95.1%)	120 (93.8%)	0.532
Yes	25 (4.9%)	8 (6.2%)	
History of alcoholism			
No	468 (91.4%)	113 (88.3%)	0.274
Yes	44 (8.6%)	15 (11.7%)	
Surgical position			
Supine	61 (11.9%)	17 (13.3%)	0.672
Prone	451 (88.1%)	111 (86.7%)	
Surgical segment			
Single	455 (88.9%)	117 (91.4%)	0.404
Multi	57 (11.1%)	11 (8.6%)	
Preoperative sleep disorder			
No	335 (65.4%)	89 (69.5%)	0.380
Yes	177 (34.6%)	39 (30.5%)	
Sevoflurane			
No	124 (24.2%)	32 (25%)	0.854
Yes	388 (75.8%)	96 (75%)	
Dexmedetomidine			
No	99 (19.3%)	31 (24.2%)	0.219
Yes	413 (80.7%)	97 (75.8%)	
Vasoactive drugs			
No	225 (43.9%)	54 (42.2%)	0.720
Yes	287 (56.1%)	74 (57.8%)	
BIS monitoring			
No	113 (22.1%)	25 (19.5%)	0.532
Yes	399 (77.9%)	103 (80.5%)	
ESPB			
No	338 (66%)	82 (64.1%)	0.677
Yes	174 (34%)	46 (35.9%)	
PONV			
No	426 (83.2%)	102 (79.7%)	0.349
Yes	86 (16.8%)	26 (20.3%)	
POD			
No	492 (96.1%)	122 (95.3%)	0.689
Yes	20 (3.9%)	6 (4.7%)	
Satisfaction of ward environment			
No	298 (58.2%)	83 (64.8%)	0.171
Yes	214 (41.8%)	45 (35.2%)	

*Mann-Whitney test

PSD: postoperative sleep disturbance, BMI: body mass index, PACU: post anesthesia care unit, CRP: C-reactive protein, VAS: visual analogue scale, SAS: self-rating anxiety scale, ASA: American society of anesthesiologists physical status classification, OSAS: obstructive sleep apnea syndrome, BIS: bispectral index, ESPB: erector spinae plane block, PONV: postoperative nausea and vomiting, POD: postoperative delirium

The day before the surgery, all patients were evaluated by researchers who collected their baseline data, such as gender, age, height, weight, past medical history, and laboratory results. The researchers also assessed the patient’s anxiety and sleep status using the SAS (Self-Rating Anxiety Scale) and PSQI. The patients were instructed on how to complete the self-assessment. During surgery and anesthesia, the researchers did not intervene and did not inform the surgeon and anesthesiologist that the patients were part of the study. Instead, the researchers collected relevant clinical data by reviewing the medical records. After the surgery, the researchers followed up with the patients for three days to monitor postoperative nausea, vomiting, and pain (the average VAS score of three nights after the operation was considered a postoperative VAS score).

### Statistical analysis

After data collection, all patients were randomly assigned to either the training or validation set in a 4:1 ratio. Normally distributed continuous variables were presented as mean ± standard deviation and compared using independent sample t-test, while non-normally distributed continuous variables were expressed as median and interquartile ranges (IQRs) and compared using the Mann-Whitney test. Dichotomous variables were reported as numbers and percentages and compared using the Chi-square test. In the training set, LASSO regression was used with PSD as the outcome variable and the 30 collected clinical variables as explanatory variables. The relationship curve between partial likelihood deviation (binomial deviation) and log (Lambda) was plotted according to 10-fold cross-validation with minimum criteria, and the optimal values were marked using the minimum and 1-SE criteria. The non-zero coefficient variables from the LASSO regression were included in the logistic regression analysis, and the independent risk factors related to PSD were determined using the stepwise forward method. Afterward, based on the selected predictors, a nomogram prediction model was formulated using the RMS package of R software, and an online dynamic nomogram was created using Shinyapps (version 0.13.2.26). To evaluate the performance of the nomogram, ROC curves, calibration plots, and DCA were used in both the training and validation sets. Statistical analysis was conducted using R software (version 4.1.2; https://www.r-project.org) and SPSS 24.0 (IBM, Chicago, IL, USA), and a two-sided P-value of less than 0.05 was considered statistically significant.

## Results

### Clinical characteristics of all patients

**Fig. 1:**
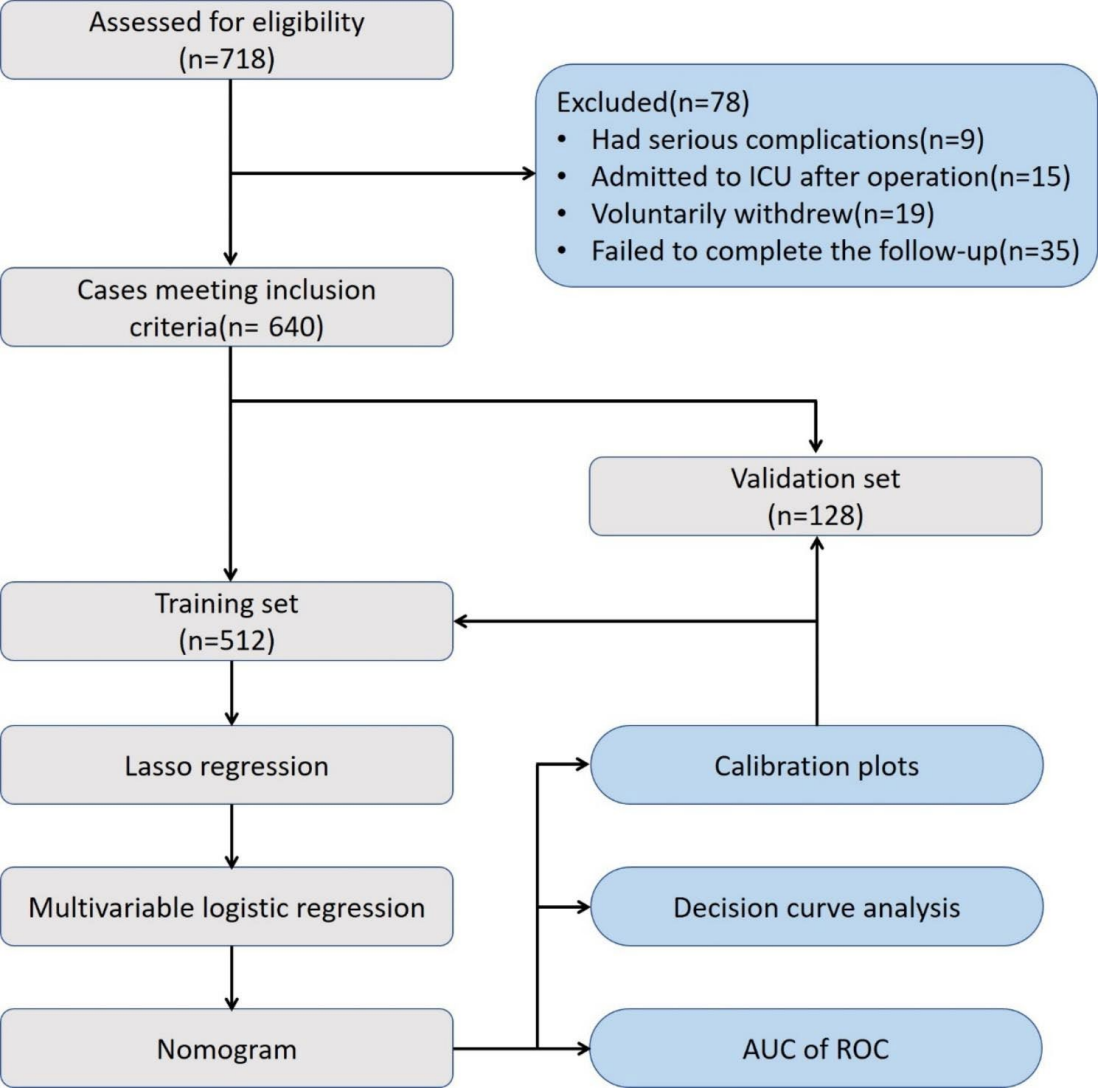
Flow chart of the study. ICU: intensive care unit, AUC: area under the curve, ROC: receiver operating characteristic curve

After excluding patients who withdrew from the study due to serious complications (9), were admitted to the ICU after the operation (15), voluntarily withdrew (19), or were lost to follow-up (35), a total of 640 patients with complete data were included in the final analysis. These patients were randomly assigned to either a training set (n = 512) or a validation set (n = 128) using a 4:1 ratio, as shown in Fig. 1.

In the training set, 314 patients (incidence of 61.3%, 314/512) were identified as having PSD based on the PSQI questionnaire evaluation, while in the validation set, 79 patients (incidence of 61.7%, 79/128) were identified as having PSD. Table 1 summarizes all the related variables, including baseline data, past medical history, laboratory findings, intraoperative treatments, drugs, and questionnaire survey results. There were no statistically significant differences between the training and validation sets in any of the characteristics (all *p*-values > 0.05).

### Construction of the nomogram

**Fig. 2:**
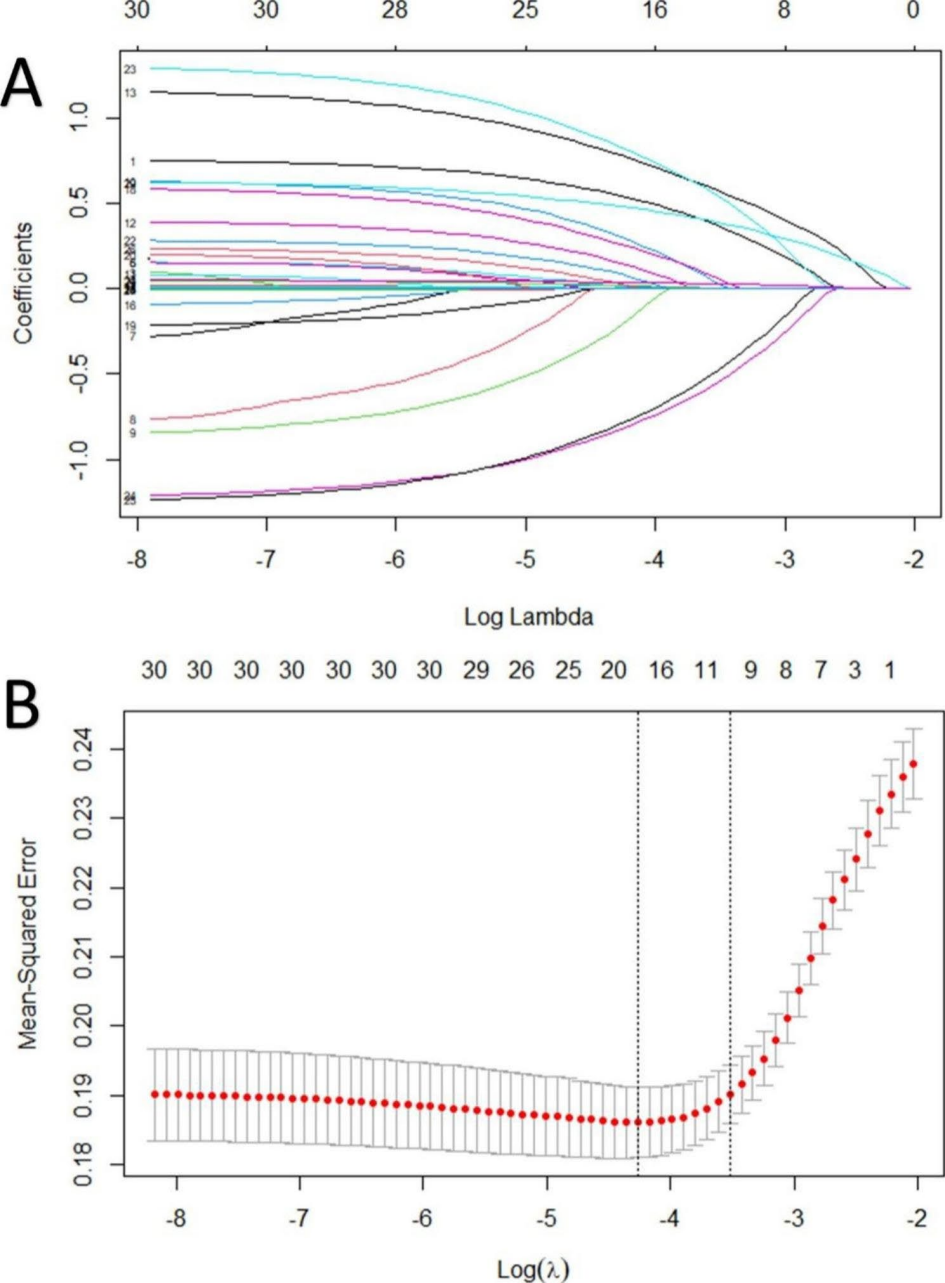
Risk factor selection using the LASSO regression model. **A**: Lasso coefficient profile plot. **B**: The result of 10-fold Cross-Validation. Dotted vertical lines on the left: the minimum values; dotted vertical lines on the right :the optimal values

Based on the results of LASSO regression (Figs. 2), 11 potential risk predictors with non-zero coefficients were identified from 30 related factors and are listed in Table 2.

**Table 2:** Coefficients of the LASSO regression model

Factors	Coefficients
Gender(female / male)	0.096
BMI(kg/m^2^)	0.002
Diabetes mellitus (Yes/ No)	0.004
Preoperative sleep disorder (Yes / No)	0.135
Bleeding volume(mL)	0.001
VAS score	0.059
SAS score	0.003
Satisfaction with ward environment (No / Yes)	0.033
Use of dexmedetomidine (Yes / No)	-0.073
Use of ESPB (Yes / No)	-0.107
Midazolam(mg)	0.001

BMI: body mass index, VAS: visual analogue scale, SAS: self-rating anxiety scale, ESPB: erector spinae plane block

Logistic regression analysis was performed on the 11 potential risk predictors selected by LASSO regression. After gradually excluding three variables through forward stepwise method, eight independent risk factors related to PSD were ultimately selected. Use the “scale” function to standardize the three continuous variables(bleeding volume, VAS score, SAS score), and then use the “lrm” function to obtain the regression coefficients and OR values of the eight variables, as shown in Table 3.

**Table 3:** **Results of logistic regression analysis**

Factors	β Coefficient	OR (95% CI)	P-Value
Gender(female / male)	0.726	2.067(1.338–3.194)	0.001
Preoperative sleep disorder (Yes / No)	0.939	2.556(1.599–4.087)	< 0.001
Bleeding volume	0.612	1.844(1.260–2.697)	0.002
VAS score	1.218	3.380(2.323–4.918)	< 0.001
SAS score	0.715	2.045(1.503–2.783)	< 0.001
Satisfaction with ward environment (No / Yes)	1.209	3.349(2.072–5.413)	< 0.001
Use of dexmedetomidine (Yes / No)	-1.171	0.310(0.174–0.553)	< 0.001
Use of ESPB (Yes / No)	-1.133	0.322(0.196–0.530)	< 0.001
Intercept	0.635	—	0.038

VAS: visual analogue scale, SAS: self-rating anxiety scale, ESPB: erector spinae plane block, OR: odds ratio

**Fig. 3:**
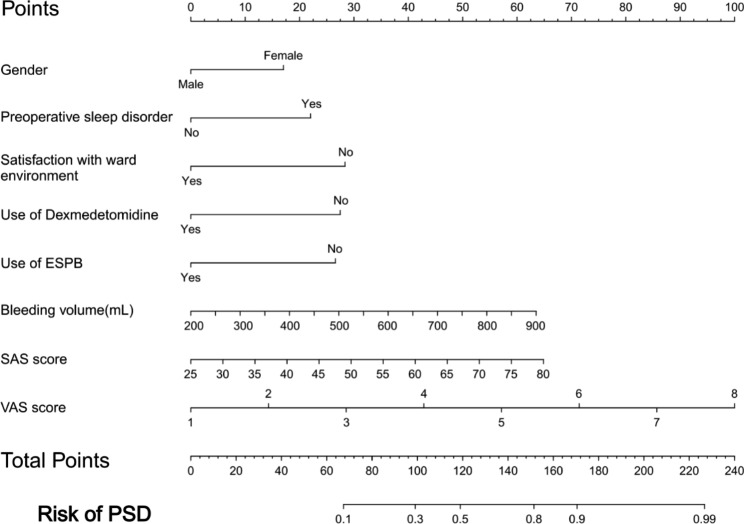
Nomogram to predict the probability of PSD in spinal surgery. Place a vertical line at the level of each variable. Add up the points for all variables and place a vertical line downwards at the corresponding total to obtain the predicted probability of PSD

Based on the regression coefficients of the eight variables, a prediction model for PSD was constructed. After restoring the continuous variables back to their original scale, a nomogram was plotted(Fig. 3).

Next, we developed an online dynamic nomogram using Shinyapps with the above-mentioned eight predictors. Clinical medical personnel can access the dynamic nomogram by visiting “https://dujin.shinyapps.io/NomforPSD/“.

**Fig. 4:**
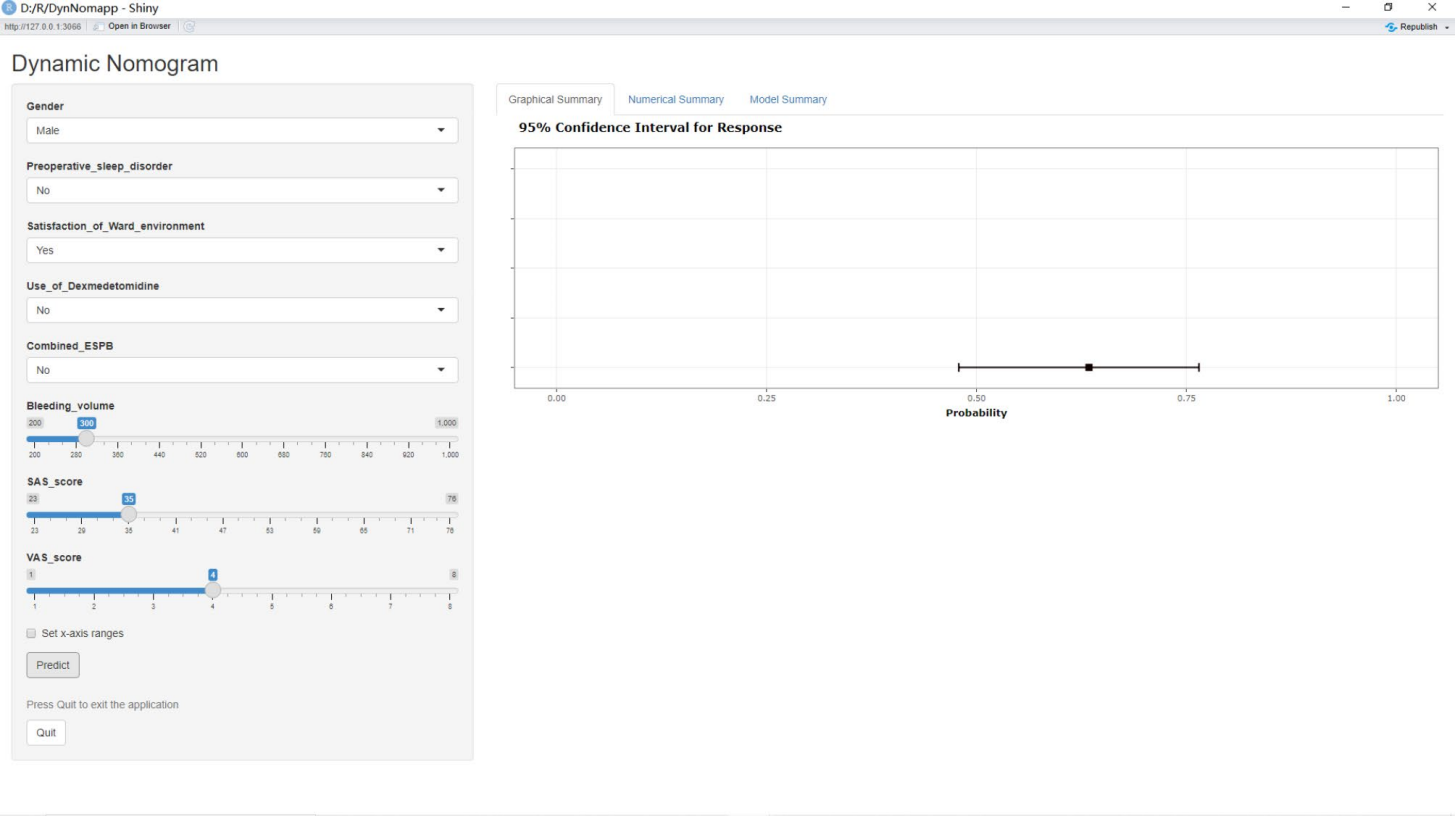
The online dynamic nomogram. After entering the patient’s data on the left side, click the “Predict” button to calculate the probability of the patient having PSD and its 95% confidence interval. https://dujin.shinyapps.io/NomforPSD/

The resulting figure (Fig. 4) displays the predicted probability of PSD [0.634 (0.479–0.635)] for a virtual patient.

### Validation of the prediction model

**Fig. 5:**
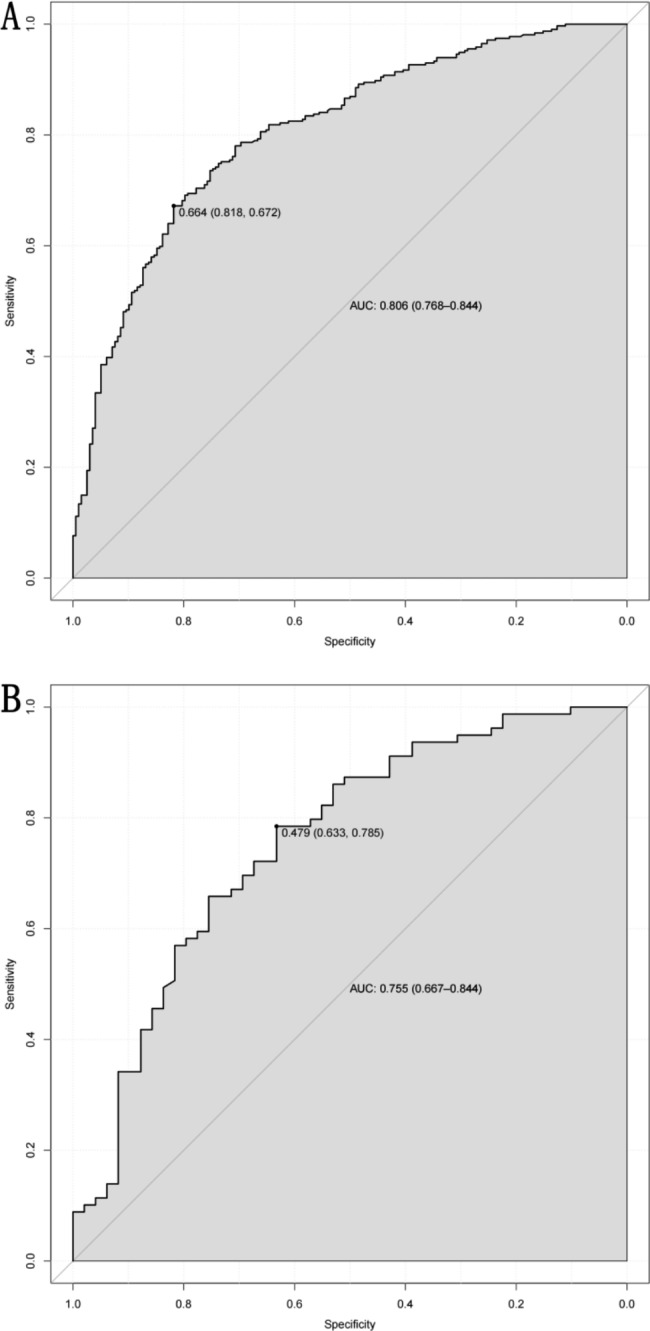
ROC curves of the nomogram for predicting the probability of PSD. **A**: the training set; **B**: the validation set. The black dot in the figure is the optimum threshold of ROC

Typically, the receiver operating characteristic (ROC) curve and area under the curve (AUC) are used to assess the precision of a prediction model. A model with a higher AUC is considered to have better predictive performance. In this nomogram model, the AUC values for the training and validation sets were 0.806 (0.768–0.844) [Fig. 5A] and 0.755 (0.667–0.844)[Fig. 5B], respectively, indicating good accuracy.

**Fig. 6:**
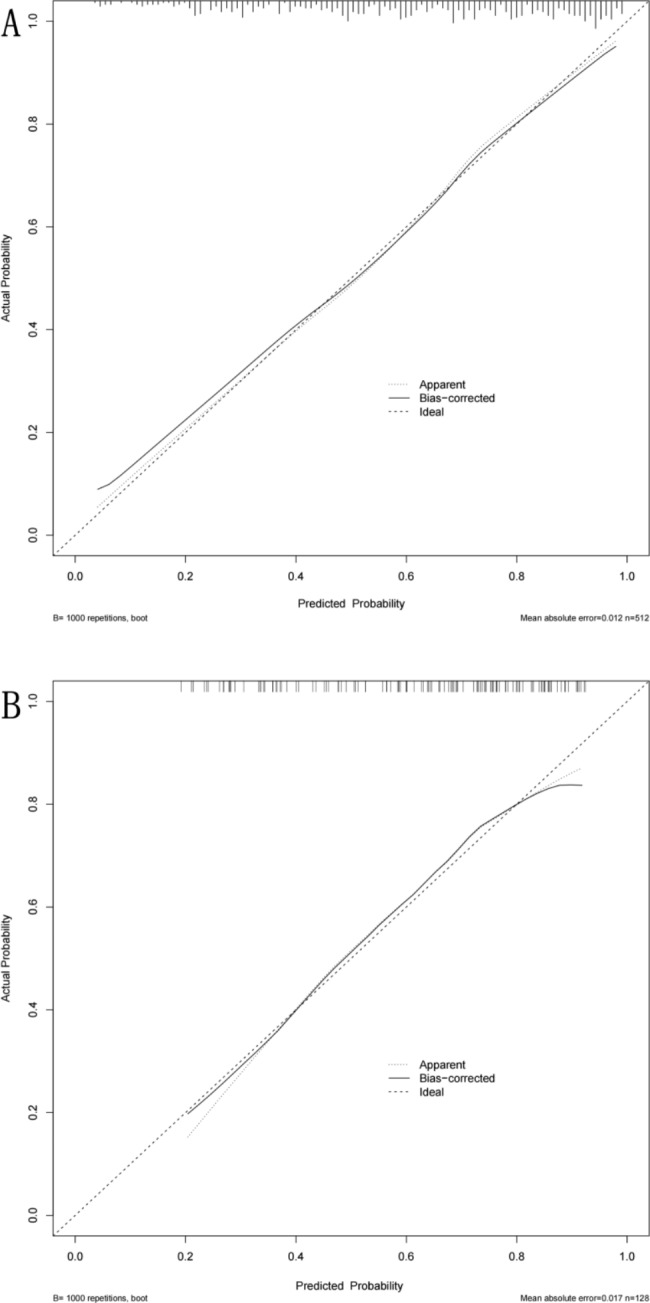
Calibration plots of the nomogram. **A**: the training set; **B**: the validation set. The dashed line represents the performance of an ideal nomogram. The dotted line represents the entire cohort. The solid line is bias-corrected by bootstrapping(B = 1000 repetitions)

A calibration plot is a tool used to compare the actual and predicted results of the nomogram. In this study, the predicted results of both the training and validation sets were in close agreement with the actual outcomes, as demonstrated in Fig. 6.

**Fig. 7:**
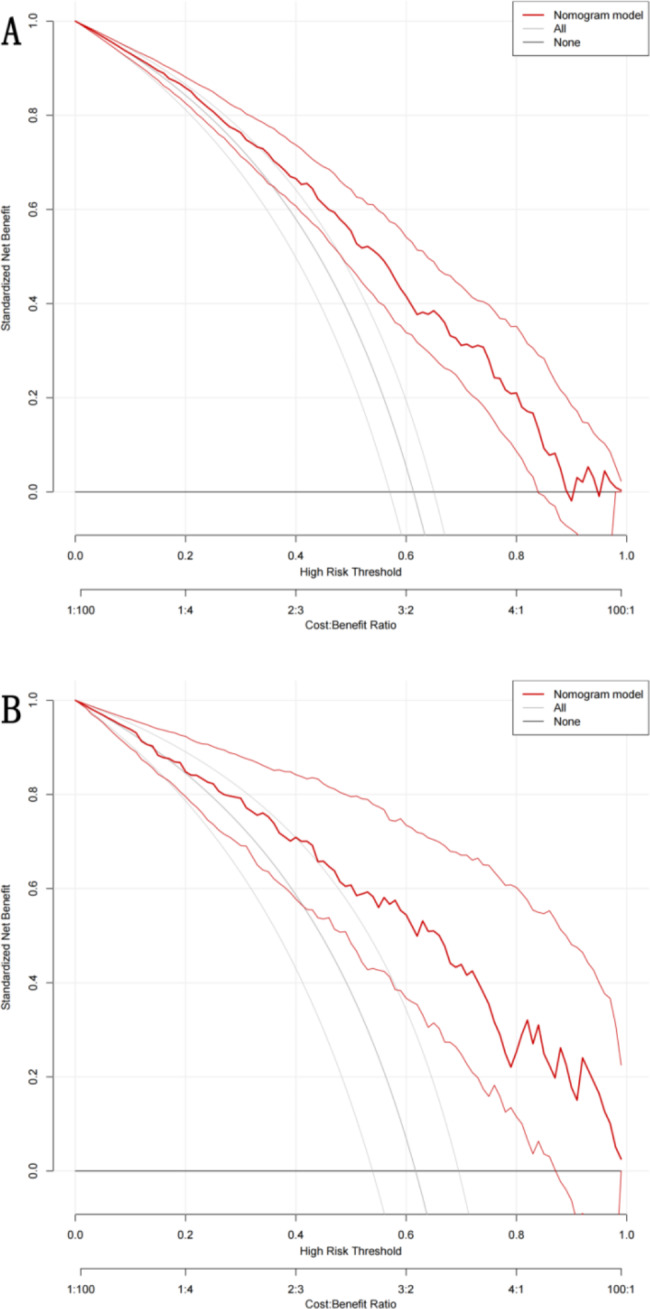
Decision curve analysis of the nomogram. **A**: the training set; **B**: the validation set. The black line(All-line) represents the assumption that intervention is performed on all patients. The gray line(None-line) represents the assumption that intervention is performed on no patients. The red line represents the nomogram. The adjacent light lines on both sides represent 95% confidence interval

The decision curve analysis (DCA) evaluates the net benefit of the nomogram prediction model at different threshold probabilities. In this study, the DCA showed that the decision curves were mostly above the ‘none’ and ‘all’ lines for most threshold ranges (20 − 90%), indicating that using the prediction model to intervene in patients provides a greater benefit than alternative strategies such as intervening in all patients, intervening in no patients, or only intervening in patients diagnosed with PSD, except for a small range of low preferences. Figure 7 illustrates the DCA results.

## Discussion

Postoperative sleep disturbance is a frequently occurring but frequently overlooked condition that has significant negative consequences, including shortened sleep duration, changes in sleep patterns, increased arousal threshold, and subsequent complications such as postoperative stress, hyperalgesia, emotional irritability, delirium, gastrointestinal dysfunction, and decreased immune responses [18, 19]. Continuous lack of sleep can lead to heightened pain sensitivity, making it difficult to fall asleep, creating a vicious cycle. Clinical treatments for PSD typically include enhancing analgesic measures (preventive analgesia, multimodal analgesia, perioperative nerve block, etc.), reducing stress responses, exogenous melatonin therapy, and improving the ward environment [20–22]. Identifying high-risk patients with PSD in advance and providing proactive interventions, such as optimizing analgesic measures, reducing the stress response, exogenous melatonin therapy, and improving the ward environment, can significantly improve postoperative sleep quality and reduce the incidence of PSD, leading to better outcomes for patients. The nomogram is an intuitive and practical prediction tool that holds significant potential for clinical applications.

In our research, we utilized LASSO regression to screen variables and adjust for complexity. We also employed logistic regression analysis to identify independent risk factors. Ultimately, we identified eight predictors and established a nomogram prediction model. As illustrated in Fig. 5, the AUC of the nomogram was 0.806 (0.768–0.844) and 0.755 (0.667–0.844) in the training and validation sets, respectively, indicating high sensitivity and specificity. Calibration curves were generated after 1000 repeated bootstrap samplings, as depicted in Fig. 6. The MAE values for both sets were 1.2% and 1.7%, respectively, indicating strong agreement between prediction and actual observation. When evaluating a model, practical value, in addition to sensitivity and accuracy, must also be considered. In clinical practice, positive intervention on patients predicted by the model will yield positive benefits, while intervention on false-positive patients will result in negative benefits. Decision curve analysis (DCA) can assess the net benefit generated by a model in clinical practice and determine its practical value. The DCA plot consists of a horizontal axis representing the threshold probability, which is the probability at which intervention will be beneficial, and a vertical axis representing net benefit, which is the benefit minus loss after intervention measures based on the model’s predicted results. As illustrated in Fig. 7, the black line (none-line) represents zero net benefit if intervention is performed on no patients, while the gray line (all-line) represents a gradual decrease in net benefit as the threshold probability increases if intervention is performed on all patients. The closer the DCA curve is to the upper right corner of the plot, away from the two extreme lines, the greater the net benefit and clinical application value. Figure 7 demonstrates that the model has a high net benefit within the range of threshold probabilities between 20% and 90%.

The nomogram we developed for predicting PSD incorporates eight clinical factors, namely gender, bleeding volume, VAS score, SAS score, preoperative sleep disorder, ward environment, dexmedetomidine, and ESPB. A prior investigation revealed that Chinese female outpatients had a significantly higher prevalence of PSD [671/2896, 23.2%] than male outpatients [302/1503, 20.1%] [23]. This disparity may be attributed to females being more sensitive to their environment and having a lower threshold for tolerating discomfort than males. Consequently, they are more susceptible to negative emotions during the perioperative period, such as irritability, anxiety, and fear, which can exacerbate PSD [23, 24]. Preoperative sleep disorders and anxiety are common risk factors that adversely affect postoperative sleep quality, and the two are interrelated. Patients frequently experience tension, anxiety, and irritability that significantly compromise the quality of their sleep due to concerns regarding procedural safety, postoperative pain, surgical efficacy, and medical costs [25, 26]. Therefore, it is imperative that medical professionals pay special attention to female patients, as well as patients with anxiety and preoperative sleep disorders. The provision of early psychological counseling and psychosocial support is crucial in alleviating patients’ emotional distress and preventing PSD.

In cases of traumatic surgery with massive intraoperative bleeding, the stress response induced by the surgery may be intensified, leading to more severe pain [17, 27]. The primary manifestation of the stress response is the activation of the hypothalamic-pituitary-adrenal (HPA) axis, leading to increased secretion of glucocorticoids, primarily cortisol. Excess cortisol secretion can result in increased production of tryptophan pyrrolidine, which lowers tryptophan concentrations in the blood. Since tryptophan is a precursor to serotonin, insufficient tryptophan levels can reduce serotonin synthesis, ultimately leading to reduced melatonin (N-acetyl-5-methoxytryptamine) secretion, shallow sleep, early awakening, poor sleep quality, and nightmares [28–30]. Conversely, massive intraoperative bleeding often indicates extreme postoperative pain, which significantly impacts patients’ postoperative sleep quality. Therefore, special attention should be given to surgical procedures that involve such interventions to mitigate trauma-related stress symptoms and reduce the likelihood of PSD.

Anesthetic drugs have a significant impact on patients’ postoperative sleep patterns. Dexmedetomidine, which acts on the α2 receptor in the nucleus locus coeruleus, induces a sedative state akin to natural sleep. Several studies have demonstrated that dexmedetomidine can enhance patients’ sleep quality after surgery [31–33]. Our study demonstrated that the use of dexmedetomidine had a protective effect against PSD (OR = 0.310, 95%CI: 0.174–0.553). Therefore, considering the potential benefits of improving postoperative sleep quality, the intraoperative administration of dexmedetomidine should be considered for surgical procedures likely to induce PSD. Previous research has shown that sevoflurane has a negative impact on postoperative sleep quality when compared to propofol, and this has been corroborated by several animal model experiments [34]. The use of sevoflurane in rats markedly suppressed the expression of Per2 (a circadian rhythm protein) in the suprachiasmatic nucleus (SCN). This mechanism is akin to the mechanism by which sevoflurane impairs postoperative sleep quality in patients [35, 36]. Our research did not establish sevoflurane as an independent risk factor for PSD, and the same was observed for sufentanil and midazolam. Nonetheless, we acknowledge the need for further investigation to confirm these findings. In clinical practice, anesthetic drugs that have the potential to affect patients’ sleep quality should be meticulously considered.

ESPB facilitates the diffusion of local anesthetics into the paravertebral space, thereby affecting the dorsal, ventral, and communicating branches of the spinal nerve [37]. Recent advancements in ultrasound technology have facilitated the safe and precise blocking of the vertical spinal muscle plane, which can alleviate postoperative pain in spinal surgery and improve patients’ postoperative sleep quality. Previous literature has shown that ESPB can reduce the dosage of anesthetic drugs used in spinal surgery and alleviate both intraoperative and postoperative pain [38–40]. Our regression analysis revealed that the use of anesthesia in combination with ESPB had a protective effect that significantly decreased the likelihood of PSD in patients undergoing spinal surgery (OR = 0.322, 95%CI: 0.196–0.530).

Postoperative pain has always been linked to the development of PSD. Pain can induce the secretion and release of various hormones, including catecholamines, glucagon, and antidiuretic hormone, via central and sympathetic nerves, leading to increased brain excitability and causing insomnia. The lack of sleep can, in turn, lead to increased pain sensitivity and difficulty in falling asleep [41, 42], creating a vicious cycle. The potential mechanisms underlying sleep deficiency and enhanced pain sensitivity primarily involve three aspects: (1) sleep deficiency causes dysfunction in the secretion and receptor function of 5-hydroxytryptamine (5-HT), reducing its pain inhibition effect; (2) sleep disorders affect the opioid receptor pathway in the brainstem and impair the pain inhibitory system; and (3) sleep disorders induce the secretion and release of various inflammatory factors that mediate pain, leading to hyperalgesia. Therefore, anesthesiologists should provide effective multimodal analgesia to alleviate postoperative pain for such patients.

The uncomfortable ward environment is often considered the primary factor that affects patients’ sleep quality [43]. Our study revealed that over half of the patients were dissatisfied with their ward environment. Patients were primarily disturbed by noise, excessively bright lighting, frequent disruptions, and environmental imbalances. Regression analysis indicated that the satisfaction level with the ward environment was a significant risk factor for PSD (OR = 3.349, 95%CI: 2.072–5.413). Therefore, it is recommended that patients be provided with a quieter and more comfortable sleeping environment to enhance their sleep quality.

Previous studies have suggested that elderly patients are more susceptible to PSD [44–46], which was not reflected in our findings. This could be because most of the patients included in our study were elderly, and the age difference was not significant enough to yield positive results. Another study reported a definite correlation between perioperative sleep quality and obstructive sleep apnea syndrome (OSAS), a common sleep-related disorder [47]. However, in our study, OSAS did not emerge as an independent risk factor, possibly due to the patients’ inability to assess whether they have OSAS, leading to imprecise results. Further extensive research is needed to establish the relationship between these risk factors and PSD.

Our nomogram model differed from other previous studies [44, 48–50], which might be attributed to differences in the target populations and types of surgeries investigated. Our study has some limitations. Firstly, the scoring method relied on self-assessment forms completed by patients, which might have been influenced by their individual understanding, cultural level, and emotional state, potentially resulting in skewed results. Future studies could use comprehensive methods such as polysomnography to assess patients’ sleep quality more objectively and accurately. Secondly, aside from the established risk factors, including anesthetic drugs, surgical trauma, and postoperative pain, many other potential factors, such as nerve root symptoms, open surgical wounds, wound drainage position, and the use of cervical braces, could contribute to PSD but were not examined in our study. Further research is necessary to investigate these crucial factors. Thirdly, the clinical data we prospectively collected has more consistency and credibility compared to retrospective studies, but it also has the disadvantage of a smaller sample size. Lastly, as this is a single-center study without external validation, there may be overfitting in the results. It is hoped that other researchers will validate the prediction model with external data to further evaluate its generalizability.

## Conclusions

Our study identified gender, preoperative sleep disorder, bleeding volume, VAS score, SAS score, ward environment, dexmedetomidine, and ESPB as predictors for PSD in patients undergoing spinal surgery. We developed a nomogram prediction model based on these eight variables, which showed good accuracy and discrimination in both the training and validation sets. This prediction model can be employed in clinical practice to identify high-risk patients for PSD beforehand and devise personalized treatment strategies for better patient outcomes.

## Data Availability

The datasets used and analyzed during the current study are available from the corresponding author on reasonable request.

## Abbreviations

PSD: Postoperative sleep disturbance
LASSO: Least absolute shrinkage and selection operator
ROC: Receiver operating characteristic curve
DCA: Decision curve analysis
VAS: Visual analogue scale
SAS: Self-rating anxiety scale
ESPB: Erector spinae plane block
AUC: Area under the curve
ASA: American society of anesthesiologists physical status classification
PSQI: Pittsburgh sleep quality index
BMI: Body mass index
ICU: Intensive care unit
PACU: Post anesthesia care unit
CRP: C-reactive protein
OSAS: Obstructive sleep apnea syndrome
BIS: Bispectral index
PONV: Postoperative nausea and vomiting
POD: Postoperative delirium
